# Brain Temperature Alters Contributions of Excitatory and Inhibitory Inputs to Evoked Field Potentials in the Rat Frontal Cortex

**DOI:** 10.3389/fncel.2020.593027

**Published:** 2020-12-07

**Authors:** Mizuho Gotoh, Kazuaki Nagasaka, Mariko Nakata, Ichiro Takashima, Shinya Yamamoto

**Affiliations:** ^1^Integrative Neuroscience Research Group, Human Informatics and Interaction Research Institute, National Institute of Advanced Industrial Science and Technology (AIST), Tsukuba, Japan; ^2^Graduate School of Comprehensive Human Sciences, University of Tsukuba, Tsukuba, Japan; ^3^Institute for Human Movement and Medical Sciences, Niigata University of Health and Welfare, Niigata, Japan

**Keywords:** temperature, focal brain cooling, frontal cortex, GABA, glutamate, antagonist, evoked potential

## Abstract

Changes in brain temperature have been reported to affect various brain functions. However, little is known about the effects of temperature on the neural activity at the network level, where multiple inputs are integrated. In this study, we recorded cortical evoked potentials while altering the local brain temperature in anesthetized rats. We delivered electrical stimulations to the midbrain dopamine area and measured the evoked potentials in the frontal cortex, the temperature of which was locally altered using a thermal control device. We focused on the maximum negative peaks, which was presumed to result mainly from polysynaptic responses, to examine the effect of local temperature on network activity. We showed that focal cortical cooling increased the amplitude of evoked potentials (negative correlation, >17°C); further cooling decreased their amplitude. This relationship would be graphically represented as an inverted-U-shaped curve. The pharmacological blockade of GABAergic inhibitory inputs eliminated the negative correlation (>17°C) and even showed a positive correlation when the concentration of GABA_A_ receptor antagonist was sufficiently high. Blocking the glutamatergic excitatory inputs decreased the amplitude but did not cause such inversion. Our results suggest that the negative correlation between the amplitude of evoked potentials and the near-physiological local temperature is caused by the alteration of the balance of contribution between excitatory and inhibitory inputs to the evoked potentials, possibly due to higher temperature sensitivity of inhibitory inputs.

## Introduction

The effects of temperature on animals’ behavior and neural activity have garnered significant attention. It has long been known that cooling focal regions of the brain inactivates their function (Brooks, 1983). Classically, cortical cooling around the central sulcus has been reported to induce a reversible inactivation of motor function (Trendelenburg, 1911). Recently, some evidence has suggested that changes in brain temperature can affect various brain functions, including responses to sensory inputs (Payne et al., 1996; Hupé et al., 1998; Lomber and Malhotra, 2008), working memory (Fuster and Bauer, 1974; Bauer and Fuster, 1976; Fuster et al., 1981), and song production in songbirds (Long and Fee, 2008; Aronov and Fee, 2012). Furthermore, brain temperature is important in a clinical context (Wang et al., 2014). Pathological neural hyperexcitability is closely related to temperature. A severe fever causes febrile convulsions in infants (Dubé et al., 2009), and focal brain cooling has been reported to be an effective treatment in epileptic patients (Ommaya and Baldwin, 1963; Karkar et al., 2002; Nomura et al., 2014).

Previous electrophysiological studies have revealed that temperature changes can also modulate neural activity (Bindman et al., 1963; Moseley et al., 1972; Moser et al., 1993; Sabatini and Regehr, 1996; Hupé et al., 1998; Schwerdtfeger et al., 1999; Aihara et al., 2001; Long and Fee, 2008; Stujenske et al., 2015; Ait Ouares et al., 2019; Owen et al., 2019). However, how this modulation occurs remains controversial. Some studies have reported that cooling the brain decreases neural activity (or that heating the brain increases neural activity; Hupé et al., 1998; Stujenske et al., 2015). It has also been reported that, during an epilepsy episode, cooling the brain could cease hyperactivity in human patients (Ommaya and Baldwin, 1963; Karkar et al., 2002; Nomura et al., 2014), as well as in animals (Motamedi et al., 2006; Inoue et al., 2017). Other studies have reported contradictory findings, i.e., that cooling the brain increases neural activity (or that heating the brain decreases neural activity; Bindman et al., 1963; Moser et al., 1993; Schwerdtfeger et al., 1999; Ait Ouares et al., 2019; Owen et al., 2019). More importantly, little is known about the effects of temperature on brain information processing at the network level, where multiple inputs of different neurotransmitters are integrated (e.g., glutamate, GABA, dopamine, et cetera).

To elucidate how changes in brain temperature affect network-level information processing in the brain, we focused on how the local brain temperature modulates cortical evoked potentials. Physiological brain temperature is typically maintained at approximately 35–39°C (Fuller, 1984; Deboer and Tobler, 1996; Kiyatkin et al., 2002; Kiyatkin, 2010; Wang et al., 2014; Vieites-Prado et al., 2016). Changes in brain temperature depend not only on changes in neural activity but also on the heat loss to the body *via* blood flow (Abrams et al., 1965; Hayward and Baker, 1969; Kiyatkin et al., 2002; Kiyatkin, 2010). Arterial blood temperature is typically lower than brain temperature (Abrams et al., 1965; Hayward and Baker, 1969; Kiyatkin et al., 2002; Kiyatkin, 2010), and an increase in blood flow decreases local brain temperature (Hayward and Baker, 1969). Magnetic resonance imaging (MRI) studies have shown that an increase in local field potentials (LFPs), but not in unit activities, is correlated with an increased blood oxygenation level-dependent (BOLD) signal (Ogawa et al., 1990; Logothetis et al., 2001). Since the BOLD signal increases when the blood flow increases (Ogawa et al., 1990), a correlation between local brain temperature and field potentials could be predicted. Based on this assumption, we recorded cortical evoked potentials while altering the local brain temperature in rats.

In this study, we examined how field potentials change when altering local brain temperature. We delivered electrical stimulations to the midbrain dopamine area and measured the evoked potentials in the frontal cortex. It is well-established that stimulating the midbrain dopamine area causes evoked responses in the frontal cortex (Mercuri et al., 1985; Lavin et al., 2005; Watanabe et al., 2009; Kunori et al., 2014; Kabanova et al., 2015; Perez-Lopez et al., 2018). Since the cortex and the midbrain are anatomically distant structures (superficial structure vs. deep structure), the cortical temperature can be altered independently of the midbrain temperature. Thus, this pathway is suitable for examining the effect of temperature in the area where evoked potentials occur (i.e., frontal cortex). We used anesthetized animals, in which the change in neural activity, metabolism, and consequent temperature fluctuation can be minimized during experiments. Here we show that focal brain cooling causes an increase in the amplitude of evoked potentials at around physiological temperature, but that pharmacological blockade of GABAergic inhibitory inputs eliminates the increase.

## Materials and Methods

### Animals

The present study included 51 male Wistar rats [295 ± 33 g, 15 ± 3 weeks old (mean ± SD), Japan SLC Inc.; Shizuoka, Japan]. All experimental procedures were performed following the National Institutes of Health Guide for the Care and Use of Laboratory Animals, and approval for this study was granted by the Animal Care and Use Committee of the National Institute of Advanced Industrial Science and Technology (AIST).

### Surgical Procedures

**Figure 1:**
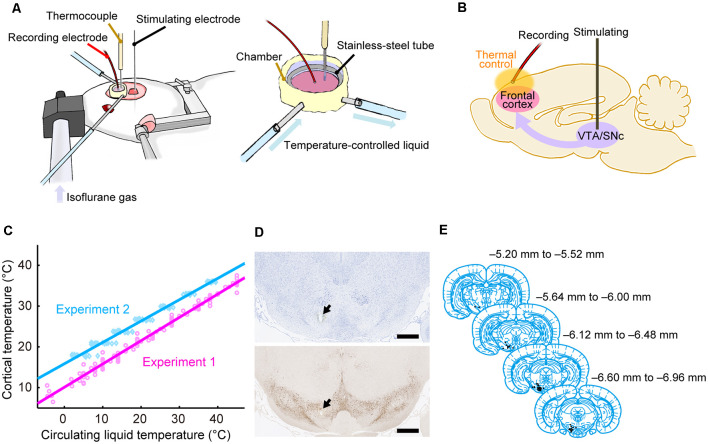
Experimental procedures. **(A)** Experimental setup using anesthetized rats. The cortical temperature was controlled by the thermal control chamber in the recording area. **(B)** Experimental system. Evoked potentials triggered by stimulation to the midbrain dopamine area were recorded from the thermal-regulated frontal cortex. **(C)** Cortical temperatures measured at a depth of 1 mm during Experiments 1 (without body temperature control; magenta circles and line) and 2 (with body temperature control; cyan diamonds and line) are plotted against the circulating water temperature. The data from all animals in Experiments 1 (*n* = 8) and 2 (*n* = 12) were fitted using linear functions (*R*^2^ = 0.98, 0.98). **(D)** Histological section of the stimulation site with Nissl staining (top) and TH staining (bottom). An electrolytic lesion was made in the midbrain dopamine area (black arrows). Scale bar, 1,000 μm. **(E)** Anatomical location of the stimulation site within the midbrain dopamine area. Histological diagrams depicting the locations of all stimulation sites corresponding to regions of the Paxinos and Watson atlas (Paxinos and Watson, 2014; adapted with permission from Elsevier Inc.; *n* = 47 animals).

Before surgery, each animal was anesthetized using 3% isoflurane gas delivered using an anesthesia device (KN-1071, Natsume Seisakusho Company, Ltd.; Tokyo, Japan) and received an intraperitoneal injection of ketamine (88 mg/kg) and xylazine (14 mg/kg). During surgery, the head of each rat was fixed using a stereotaxic device (SR-6R-HT, Narishige; Tokyo, Japan). Craniotomy was performed to make two holes in the skull; one was used to record evoked potentials in the dorsal frontal cortex and the other was used to stimulate the midbrain dopamine area [the lateral part of the ventral tegmental area (VTA), the medial part of the substantia nigra pars compacta (SNc), and the area in-between]. The circular hole for recording was centered approximately 2.5 mm anterior to the Bregma and 2.5 mm lateral to the midline on the left side, and its diameter was approximately 5 mm. The hole for SNc/VTA stimulation was located 6.8 mm posterior to the Bregma and 1.5 mm lateral to the midline on the left side; the hole was approximately rectangular (3 × 4 mm). The dura matter was removed from each area (Figures 1A,B).

### Thermal Control Device

The cortical temperature was controlled using a thermal control chamber (Figure 1A) made of dental cement (PANAVIA™ V5, Kuraray Noritake Dental Inc.; Tokyo, Japan). A coil-shaped stainless-steel tube (inner diameter = 6 mm) was embedded inside the chamber. The chamber was filled with 100 μl saline (Experiments 1 and 2, and control conditions in Experiments 5–7) or saline containing antagonists (antagonist conditions in Experiments 3–7) to control the temperature of the cortex. Using a liquid circulator (LTCi-150HP or MCX-250, As One Corporation; Osaka, Japan), temperature-controlled water flowed through the stainless-steel pipe to regulate the temperature of the cortex *via* thermal conduction. We used an anti-freeze solution instead of water when the temperature of the circulating liquid had to be less than 0°C in Experiments 1 and 3. During experiments, the cortical temperature was recorded with a thermometer (BAT-10R/LOP, Physitemp Instruments, Inc.; Clifton, NJ, USA) by inserting a thermocouple electrode (MT-29/2, Physitemp Instruments, Inc.; Clifton, NJ, USA) at a depth of 1 mm in the frontal cortex. The cortical temperature at this depth was confirmed to be linearly correlated with the temperature of the water circulating in the pipe (Figure 1C). The cortical temperature was changed before each recording. The change in cortical temperature, the interval, and the average rate of temperature change between two recordings were 3.7 ± 1.5°C, 4.2 ± 2.9 min, and 1.6 ± 1.6°C/min, respectively, in Experiments 1 and 3, 3.0 ± 0.2°C, 6.1 ± 3.7 min, and 0.6 ± 0.2°C/min, respectively, in Experiments 2 and 4, and 9.0 ± 0.3°C, 10.7 ± 8.6 min, and 1.1 ± 0.4°C /min, respectively, in Experiments 5–7 (Mokrushin et al., 2014).

### Electrophysiology

During electrophysiological experiments, each rat was kept under anesthesia using 0.5–2.5% isoflurane gas delivered *via* an anesthetic mask (GM-3, Narishige; Tokyo, Japan), and the head of each rat was fixed using a stereotaxic device (SR-6R-HT, Narishige; Tokyo, Japan). We tried to keep the concentration of isoflurane constant within each condition to minimize the effect of anesthesia concentration (Sebel et al., 1986). However, we increased the isoflurane concentration because of the decline in the depth of anesthesia in 12 of the 136 conditions (in 2 of 8, 2 of 12, 0 of 8, 1 of 12, 1 of 24, 4 of 36, and 2 of 36 conditions from Experiments 1, 2, 3, 4, 5, 6, and 7, respectively). The change in concentration within a single experimental condition was 0.052 ± 0.17% (mean ± SD). Evoked potentials on the frontal cortex were recorded using a silver ball electrode (UL-3010, Unique Medical Company, Ltd.; Tokyo, Japan) placed on the cortical surface in the thermal control chamber and filtered at a band-pass of 0.1–1,000 Hz. The ball electrode was located ~2 mm anterior to the Bregma, ~1.5 mm lateral to the midline, and ~2 mm away from the thermocouple electrode. While recording the evoked potentials on the cortex, a single electrical pulse (200 μA in amplitude, 300 μs in duration) was delivered to the midbrain dopamine area (VTA/SNc) using a concentric bipolar electrode (IMB-9002, Inter Medical Company, Ltd.; Nagoya, Japan), which was targeted to a region 5.6–7.2 mm posterior to the Bregma, 1.0–1.5 mm lateral to the midline, and at a depth of 6.5–8.5 mm (Figures 1D,E). At each temperature, evoked potentials were recorded in 10–30 trials in Experiments 1 and 3, and 30 trials in Experiments 2, 4, 5, 6, and 7. Each trial was separated by intervals of 10 s in Experiments 1 and 3, and 9.9 s in Experiments 2, 4, 5, 6, and 7. In Experiments 2, 4, 5, 6, and 7, the body temperature of the animal was controlled so that the rectal temperature was maintained at 36°C using a body-temperature-keeping device (BWT-100A, BioResearch Center; Nagoya, Japan). A custom recording system with LabVIEW 7.1 software (National Instruments; Austin, TX, USA) was used to acquire data in Experiments 1 and 3, while a multifunction processor (RX6; Tucker-Davis Technologies, Alachua, FL, USA) was used in Experiments 2, 4, 5, 6, and 7.

### Experiment 1: Effects of Brain Temperature Without Body Temperature Control

Eight rats were used in Experiment 1. Their body temperature was not controlled. The evoked potentials on the frontal cortex were recorded at various cortical temperatures by changing the temperature of the water circulating through the tube embedded inside the chamber. The cortical temperature without (i.e., before) thermal regulation (no saline filling the chamber) was ~27.2°C (the average of five rats before control conditions). During the experiment, the cortical temperature was experimentally controlled. The circulating water temperature started at 28°C, decreased to reach its minimum, increased to reach its maximum, and then decreased to 28°C. The ranges in circulating water temperature and in resulting cortical temperature were −4.5–45°C and 6.5–37.5°C, respectively.

No symptoms of hypothermia, such as shivering, were observed during Experiments 1 and 3. However, the cortical temperature without its regulation in Experiment 1 (~27.2°C) was ~5°C lower than the cortical temperature when the body temperature was controlled (see “Experiment 2” section ~32.3°C with a rectal temperature of 36°C). Although we did not measure the rectal temperature directly in Experiments 1 and 3, the body temperature may have decreased. In addition, focal cortical cooling may have resulted in a further decrease in body temperature in Experiments 1 and 3. Therefore, we controlled the body temperature in Experiments 2, 4, 5, 6, and 7 to eliminate the possibility that the decrease in the body temperature or hypothermia would affect neural activity, metabolism, and cardiopulmonary function.

### Experiment 2: Effects of Brain Temperature Under Body Temperature Control

Twelve rats were used in Experiment 2. The evoked potentials were recorded with their body (rectal) temperature maintained at 36°C using a body-temperature-keeping device. The cortical temperature without thermal regulation was ~32.3°C while maintaining body temperature (the average of 37 rats before control conditions in Experiments 2, 5, 6, and 7), which was equivalent to that observed in a previous study with anesthetized rats (Schwerdtfeger et al., 1999). During the experiment, the cortical temperature was precisely controlled. The cortical temperature was modified within the range of 18–36°C by adjusting the temperature of the circulating water. In six rats, the cortical temperature was increased from 18 to 36°C in 3°C steps. In the other six rats, it was decreased from 36 to 18°C in 3°C steps.

### Experiment 3: Administration of GABA_A_ Receptor Antagonist Without Body Temperature Control

The same eight rats from Experiment 1 were used in Experiment 3. We examined the effects of gamma-aminobutyric acid (GABA) on the evoked potentials by administering a GABA_A_ receptor antagonist in the recording chamber. After completion of Experiment 1, the saline was removed from the chamber, and 1 mM (in saline) of the GABA_A_ receptor antagonist SR-95531 (gabazine, Toronto Research Chemicals; Toronto, ON, Canada) was administered. After administering the antagonist, an incubation of approximately 1 h was performed for the antagonists to infiltrate within the local cortical region and to be in equilibrium in the cortex. The drug concentration that we administered was not the drug concentration that the neurons were exposed to because the antagonist molecules permeated into the cortex. The possibility of the concentration change in the chamber solution being due to evaporation could not be excluded either. During the incubation period, the cortical temperature was maintained at ~26°C. After the incubation, the evoked potentials were recorded following the procedures used in Experiment 1. The circulating water temperature and the controlled cortical temperature ranged from −4.8 to 45°C and from 4.1 to 37.6°C, respectively.

### Experiment 4: Administration of GABA_A_ Receptor Antagonist Under Body Temperature Control

The 12 rats from Experiment 2 were used in Experiment 4. Here, we also examined the effects of GABA on evoked potentials while maintaining body temperature. After Experiment 2, the saline was removed from the chamber and 1 mM gabazine was administered. Approximately 1 h after administration at ~33°C of cortical temperature, evoked potentials were recorded following the procedures used in Experiment 2.

### Experiment 5: Effects of Different Concentrations of GABA_A_ Receptor Antagonist

Six rats were used in Experiment 5. We examined the dose-dependency of the effect of gabazine on the evoked potentials. The cortical temperature was altered within the range of 18–36°C in 9°C steps (18°C, 27°C, and 36°C) while maintaining a body temperature of 36°C. The change in cortical temperature was conducted in ascending order in three rats, and in descending order in the other three rats. In the control, the evoked potentials were recorded with the chamber filled with saline. For the second condition, 10 μM gabazine was administered to the chamber after removing the saline. Approximately 1 h later, the evoked potentials were recorded. For the third and fourth conditions, 100 and 1,000 μM gabazine was administered in the chamber. During the incubation period (~1 h), the cortical temperature was maintained at 33°C.

### Experiment 6: Administration of Glutamate Receptor Antagonists

Twelve rats were used in Experiment 6. We examined the effects of glutamate on the evoked potentials by administering its antagonists. The cortical temperature was modified within the range of 18–36°C in 9°C steps while maintaining the body temperature at 36°C. In the first condition (control), the evoked potentials were recorded with the chamber filled with saline. In the second condition, after removing the saline from the chamber, one of the two antagonists (antagonist #1) was administered: the antagonist for the α-amino-3-hydroxy-5-methyl-4-isoxazolepropionic acid (AMPA) receptor or the antagonist of the *N*-methyl-D-aspartate (NMDA) receptor. NBQX disodium salt (NBQX; Tocris Bioscience; Bristol, UK) and (R)-CPP (Tocris Bioscience; Bristol, UK) were used as antagonists of AMPA and NMDA receptors, respectively (10 mM dissolved in saline). Approximately 1 h after administering the antagonist, the evoked potentials were recorded. In the third condition, after removing the antagonist #1 solution from the chamber, the other antagonist (antagonist #2) was administered, and the evoked potentials were recorded ~1 h later. During the incubation period, the cortical temperature was maintained at 33°C. The order of temperature change (ascending vs. descending) and the order of antagonist administration (AMPA followed by NMDA vs. NMDA followed by AMPA) were balanced (i.e., three rats per each combination). One rat was excluded from the analyses (temperature: ascending, antagonist: NMDA followed by AMPA) because the stimulating electrode was positioned outside of the midbrain dopamine area (see “Histological Analysis” section).

### Experiment 7: Administration of Dopamine Receptor Antagonists

Twelve rats were used in Experiment 7, in which we examined the effects of dopamine on the evoked potentials. The procedures were essentially the same as those in Experiment 6, but two antagonists to dopamine receptors were used. One was SCH 23390 hydrochloride (SCH 23390; Tocris Bioscience; Bristol, UK), the D_1_/D_5_ receptor antagonist, and the other was raclopride (Tocris Bioscience; Bristol, UK), the D_2_/D_3_ receptor antagonist (1 mM dissolved in saline). In the first condition (control), the evoked potentials were recorded while the chamber was filled with saline. In the second condition, the evoked potentials were recorded after administering one of the dopamine receptor antagonists (antagonist #1). In the third condition, the evoked potentials were recorded after administering the other dopamine receptor antagonist (antagonist #2). During the incubation period (~1 h), the cortical temperature was maintained at 33°C. The order of temperature change (ascending vs. descending) and the order of antagonist administration (D_1_/D_5_ followed by D_2_/D_3_ vs. D_2_/D_3_ followed by D_1_/D_5_) were balanced (i.e., three rats per each combination). Two rats were excluded (one from temperature: ascending, antagonist: D_2_/D_3_ followed by D_1_/D_5_, and one from temperature: descending, antagonist: D_1_/D_5_ followed by D_2_/D_3_) because the stimulating electrode was positioned outside of the midbrain dopamine area (see “Histological Analysis” section).

### Preparation of Brain Tissues for Histological Analysis

On completion of the experiments, an electrolytic lesion was made (200 μA, 5 s) at the stimulation site. Next, an intraperitoneal injection of pentobarbital sodium (64.8 mg, Kyoritsu Seiyaku Corporation; Tokyo, Japan) was administered to induce euthanasia. All rats were perfused through the left cardiac ventricle with saline followed by a formalin solution [neutral buffered (10%), Sigma–Aldrich; St. Louis, MO, USA]. The brains were then removed and post-fixed in the same fixative over 3 days at 4°C. Next, the brains were washed with 100 mM phosphate buffer (PB) and then cryoprotected in 30% sucrose in 100 mM PB. Subsequently, some brains were frozen in isopentane (Wako Pure Chemical Industries, Ltd.; Osaka, Japan) and sliced into coronal sections (40 μm thick for Experiments 1 and 3, 30 μm thick for four rats in Experiments 2 and 4) using a sliding microtome. The other brains (i.e., from the other eight rats in Experiments 2 and 4 and all rats in Experiment 5–7) were embedded in paraffin and sliced into coronal sections of 8 μm thickness. The sections of each rat in Experiments 2, 4, and 5–7 were divided into two series. One section series from these experiments and all sections from Experiments 1 and 3 were processed for Nissl staining with 0.1–0.2% thionin blue solution. The other section series was subject to immunohistochemistry. Paraffin-embedded sections were deparaffinized and rehydrated before staining.

### Immunohistochemistry

Immunohistochemistry for tyrosine hydroxylase (TH) was conducted to identify dopamine neurons. Deparaffinized sections were incubated in 10 mM, pH 6.0 sodium citrate buffer for 30 min at 90°C. After being cooled at room temperature (RT), sections were washed and incubated with methanol and 0.3% H_2_O_2_ for 30 min at RT. Next, they were blocked with 4% Block-Ace (DS Pharma Biomedical Company, Ltd.; Osaka, Japan) in phosphate-buffered saline (PBS) containing 0.2% Triton X-100 (PBS-X) for 2 h at RT. Free-floating sections were first incubated in PBS-X with 1% H_2_O_2_ for 20 min, washed, and blocked by 3% normal goat serum in PBS-X for 2 h at RT. After the blocking procedures, both sets of sections were incubated with an anti-TH rabbit polyclonal antibody (1:1,000; AB152, Merck Millipore; Billerica, MA, USA) diluted in blocking solution at 4°C overnight. The sections were then washed and incubated with biotinylated goat anti-rabbit IgG secondary antibody (1:400; BA-1000, Vector Laboratories; Burlingame, CA, USA) for 2 h at RT. They were washed again and incubated with the avidin-biotin complex (VECTASTAIN ABC Elite kit, Vector Laboratories; Burlingame, CA, USA) in PBS for 45 (free-floating) or 30 min (paraffin-embedded) at RT. They were incubated with 0.02% DAB and 0.003% H_2_O_2_ in PBS for 2.5 min, washed, and the free-floating sections were then mounted on slides. Finally, sections were dehydrated, cleared with xylene, and cover-slipped with mounting medium.

### Histological Analysis

Based on the Nissl and TH-stained sections (Figure 1D), we identified the location of the electrolytic lesion in each brain. The stimulation site was identified in the midbrain dopamine or adjacent area in 47 of 50 rats, which were used in Experiments 1–7. We thus analyzed the data from these 47 rats. The locations of stimulation sites were mapped on coronal planes from the brain atlas (Paxinos and Watson, 2014; Figure 1E).

### Data Analysis

All data analyses were conducted using MATLAB (R2013b, The MathWorks, Inc.; Natick, MA, USA). We analyzed the evoked potential data between −40 and 250 ms (Experiments 1 and 3) or −1,000 and 250 ms (Experiments 2, 4, 5, 6, and 7) from the onset of the electrical stimulation (0 ms). The average of evoked potentials between −20 and 0 ms (Experiments 1 and 3) or −50 and 0 ms (Experiments 2, 4, 5, 6, and 7) was defined as the baseline (0 mV) for each experimental block (10–30 trials). The preliminary analysis showed that the evoked potentials were disturbed if spontaneous activity appeared close to or during the evoked potential. In that case, evoked potentials were sometimes absent, and even when they occurred, the amplitude was smaller. Because the electrical stimulations were automatically delivered with a constant interval, the spontaneous activity overlapped with the stimulation pulse or its prior period. To evaluate the effect of temperature as precisely as possible, the data from the trials in which spontaneous activity occurred 1,000 ms before the stimulation were excluded from the analyses in Experiments 2, 4, 5–7. For Experiments 1 and 3, since we only collected data 40 ms before the stimulation, trials in which evoked responses were absent as well as those with spontaneous activity occurring within 40 ms of the stimulation were excluded from the analysis. In total, we used 15,439 of 18,268 trials for the analysis.

For quantitative analyses, we identified the peak value of each evoked potential waveform. We first identified the peak at 27°C in the control as a local minimum of the waveform 10–100 ms after the stimulation. We then identified the corresponding peaks in the other conditions. We defined two indices: the peak amplitude and the peak latency. The peak amplitude was defined as the absolute (i.e., inversed) value of the voltage at the peak. If the peak was undetectable or did not reach the baseline (i.e., the peak amplitude was less than 0), the peak amplitude was defined as 0 mV. The peak latency was defined as the period from the stimulation onset to the peak. If the peak was undetectable, the peak latency remained undefined (NaN value in MATLAB).

To examine how the peak amplitudes and latencies were affected by the cortical temperature and the antagonists, we performed multiple regression analyses. The hypothesized linear model was as follows:

$$P\;=\;\beta_{0}\;+\;\beta_{a}\;\cdot \;Animal\;+\;\beta_{c}\;\cdot \;Condition\;+\;\beta_{t}\;\cdot \;(T\;-\;T_{0})\;+\;\beta_{i}\;\cdot \;(Condition\;\times \;(T\;-\;T_{0}))\;+\;\varepsilon$$

*P* denotes the peak amplitude or latency. *Animal* is a categorical variable that denotes the individual animal. For example, animal #1 is indicated as (0, 0, …, 0) and animal #3 is indicated as (0, 1, 0, …, 0). *Condition* is also a categorical variable that denotes the experimental conditions (e.g., control, 10, 100, and 1,000 μM in Experiment 5). The dimension of categorical variables corresponds to the number of categories in each experiment minus 1. *T* denotes the temperature (e.g., 18, 27, and 36°C in Experiment 5). In Experiments 1 and 3, the averaged cortical temperature was used. To estimate the effects at a physiologically normal temperature, we also set the parameter *T*_0_ as 36°C. β_0_, β_a_, β_c_, β_t_, and β_i_ denote the intercept at *T* = *T*_0_, the regression coefficient for *Animal*, the regression coefficient for *Condition*, the regression coefficient for (*T* − *T*_0_), and the regression coefficient for the interaction term between *Condition* and (*T* − *T*_0_), respectively. ε denotes the residual error of the fitted model.

The meanings of the estimated regression coefficients depend on which condition is assigned to the zero-vector for the parameter *Condition*. For example, in Experiment 5, if *Condition* = (0, 0, 0) for the control, the model estimates three values of β_c_ that correspond to the respective effects of 10 μM gabazine, 100 μM gabazine, or 1,000 μM gabazine from the control condition. If *Condition* = (0, 0, 0) for 100 μM gabazine, the model estimates three values of β_c_ that correspond to the effects of the control, 10 μM gabazine, or 1,000 μM gabazine from 100 μM gabazine. To estimate the regression coefficients for all combinations of conditions, we repeated the estimation while altering the assignment of elements in the categorical variables.

**Figure 2:**
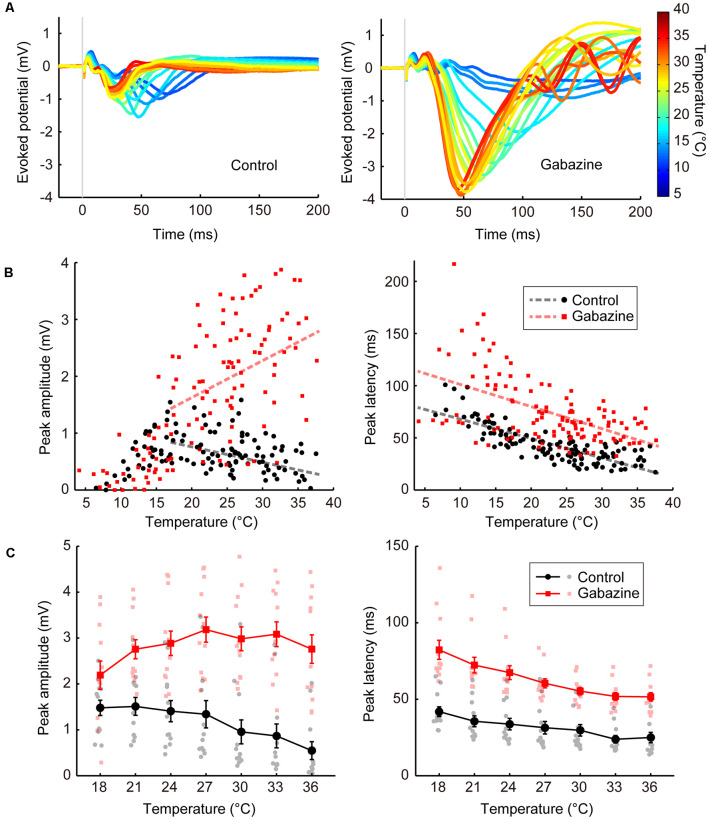
Temperature dependency of the evoked potentials and effects of GABAergic inputs (Experiments 1–4). **(A)** Representative waveforms of the evoked potentials at various temperatures (*n* = 1 animal) for the control condition (left) and the condition in which gabazine was administered (right). The evoked potentials for each cortical temperature are represented by different colors that can be identified in the rightmost color bar. **(B)** Effects of cortical temperature in the control condition (Experiment 1) and under GABA_A_ receptor antagonist administration (Experiment 3) without body temperature regulation. The peak amplitude (left) and the peak latency (right) in Experiments 1 (black circles and lines) and 3 (red squares and lines) are plotted against cortical temperature (*n* = 8 animals). Dashed lines indicate regression lines. **(C)** Effects of cortical temperature in the control condition (Experiment 2) and under GABA_A_ receptor antagonist administration (Experiment 4) with body temperature regulation. The cortical temperature was modified within the range of 18–36°C in 3°C steps while body temperature was maintained at 36°C. The peak amplitude (left) and the peak latency (right) in Experiments 2 (black circles and lines) and 4 (red squares and lines) are plotted against cortical temperature (*n* = 12 animals). Error bars indicate mean ± SE.

In Experiment 1, the peak amplitude and temperature exhibited an inverted-U relationship (Figure 2B, left, black circles). The smoothed curve (calculated with “rlowess” function in MATLAB) showed the maximum amplitude at the temperature of 17.0°C. Thus, we separately performed the multiple regression analyses with the data of >17°C and <17°C in Experiments 1 and 3. For the other experiments, we performed multiple regression analyses with the data of all temperatures.

After estimating coefficients, we calculated t-statistics for each coefficient. The null hypothesis was that the coefficient was equal to zero given the other predictors in the model. We then calculated the *P*-value (two-sided). For each multiple regression analysis, we used a Kolmogorov–Smirnov test (Massey, 1951) to confirm that the distribution of residuals of the fitted model did not significantly deviate from a Gaussian distribution.

## Results

### Temperature Dependency of Evoked Potentials

First, we examined the basal level of temperature dependency of neural activity without modifying neurotransmission. We controlled the temperature of the frontal cortex using a thermal control chamber (Figure 1A) and recorded the cortical field potentials evoked by electrical stimulation to the midbrain dopamine area (VTA/SNc; Figures 1B,D,E). The thermal control system precisely regulated the cortical temperature (Figure 1C). In Experiment 1, body temperature was not controlled. Waveforms of the evoked potentials changed according to the cortical temperature (Figure 2A, left). The relationship between the temperature and peak amplitude of the evoked potentials was non-monotonic, with the peak amplitude increasing as temperature decreased (negative correlation) until 17.0°C (β_t_ = −0.027, *P* = 0.0072, >17°C; Figure 2B, left; black circles and line). Further cooling decreased the amplitude (positive correlation; β_t_ = 0.088, *P* = 3.5 × 10^–6^, <17°C), which approached zero as the temperature dropped below 10°C (Supplementary Tables 1, 2). The peak latency was lower (faster peaks) at higher temperatures and higher (slower peaks) at lower temperatures (β_t_ = −1.862, *P* = 3.1 × 10^–16^; Figure 2B, right; black circles and line; Supplementary Table 3).

In Experiment 1, we demonstrated the dependency of neural activity on brain temperature without body temperature regulation. In Experiment 2, a heating pad was introduced to maintain the body temperature at 36°C to eliminate any effect of body temperature decline and fluctuation by anesthesia. The cortical temperature before regulating the cortical temperature (no saline filling the chamber) was ~27.2°C in Experiment 1 and ~32.6°C in Experiment 2. The amplitude increased as the temperature decreased (Figure 2C, left; black circles and lines; β_t_ = −0.054, *P* = 1.8 × 10^–5^), as in Experiment 1 (Supplementary Table 4). The peak latency was negatively correlated with temperature (Figure 2C, right; black circles and lines; β_t_ = −0.932, *P* = 3.1 × 10^–10^; Supplementary Table 5). These results show that the effects of local brain temperature were qualitatively equivalent irrespective of the presence of body temperature control, suggesting that the observed temperature dependency of evoked potentials was primarily dependent on the focal cortical temperature.

### Effects of Blocking GABAergic Inputs on the Temperature Dependency of Evoked Potentials

Second, we investigated the effects of blocking GABAergic inhibitory inputs on the temperature dependency of neural activity. We applied a GABA_A_ receptor antagonist (gabazine; 1,000 μM) to the cortices of the same rats that were used in Experiment 1 (Experiment 3; no body temperature regulation; Figure 2A, right). Amplitudes were generally higher after gabazine administration (β_c_ = 2.341, *P* = 2.1 × 10^–34^, >17°C; Figure 2B, left; red squares and line). Surprisingly, the temperature dependency was significantly altered (β_i_ = 0.092, *P* = 3.5 × 10^–10^, >17°C) such that a positive correlation was observed between peak amplitude and temperature (β_t_ = 0.065, *P* = 2.1 × 10^–10^, >17°C). Similar results were obtained when controlling body temperature at 36°C (Experiment 4). Amplitudes were higher after gabazine administration (β_c_ = 2.425, *P* = 1.6 × 10^–26^; Figure 2C, left; red squares and lines), the temperature dependency was significantly altered (β_i_ = 0.083, *P* = 3.4 × 10^–6^), and the negative correlation was eliminated to turn into slightly positive (β_t_ = 0.029, *P* = 0.018). Therefore, GABAergic inhibitory inputs are critical for establishing the negative correlation observed in the control condition (Supplementary Tables 1, 2, 4). Gabazine administration increased the peak latency (Experiment 3: β_c_ = 26.534, *P* = 1.2 × 10^–8^; Experiment 4: β_c_ = 24.256, *P* = 2.5 × 10^–22^), maintaining the negative correlation (Experiment 3: β_t_ = −2.120, *P* = 2.4 × 10^–21^, Experiment 4: β_t_ = −1.729, *P* = 4.0 × 10^–25^; Figures 2B,C, right; Supplementary Tables 3, 5).

**Figure 3:**
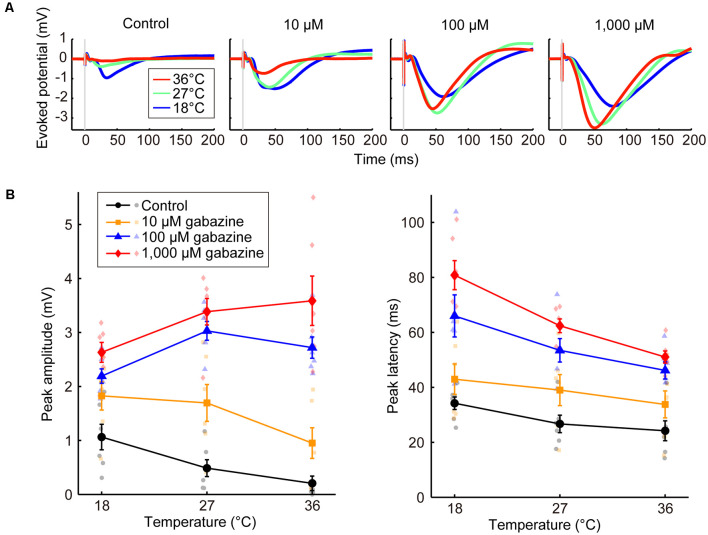
Effects of the concentration of a GABA_A_ receptor antagonist on temperature dependency (Experiment 5). **(A)** Averaged waveforms of evoked potentials. The concentrations of gabazine were 0 (control), 10, 100, and 1,000 μM. Red, green, and blue lines indicate the averaged waveforms (*n* = 6 animals) at temperatures of 36, 27, and 18°C, respectively. **(B)** Temperature dependency of the peak amplitude (left) and the peak latency (right) of the evoked potentials for the control condition (black circles and lines) and gabazine at 10 μM (yellow squares and lines), 100 μM (blue triangles and lines), and 1,000 μM (red diamonds and lines) conditions (*n* = 6 animals) are plotted against cortical temperature. Error bars indicate mean ± SE.

We also demonstrated that the effects of temperature on evoked potentials were dependent on the gabazine concentration (Experiment 5, Figure 3). Amplitude progressively increased as the gabazine concentration increased, finally showing a positive correlation between temperature and amplitude at 1,000 μM gabazine (β_t_ = 0.053, *P* = 0.0069), whereas they were negatively correlated in the control (β_t_ = −0.048, *P* = 0.015; Figure 3B, left; Supplementary Table 6). Latency also changed in a dose-dependent manner (Figure 3B, right; Supplementary Table 7).

### Effects of Blocking Glutamatergic and Dopaminergic Inputs on the Temperature Dependency of Evoked Potentials

**Figure 4:**
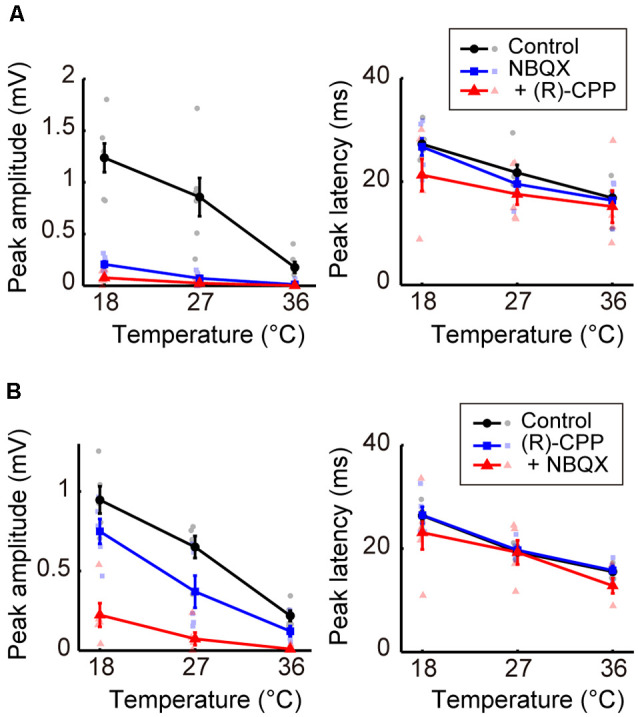
Effects of blocking glutamatergic inputs on the temperature dependency of evoked potentials (Experiment 6). **(A)** Temperature dependency when NBQX was administered first, and (R)-CPP second (*n* = 6 animals). The peak amplitude (left) and the peak latency (right) of the evoked potentials are plotted against cortical temperature. Black, blue, and red symbols and lines indicate data for the control, for when one antagonist was administered, and for when two antagonists were administered, respectively. Error bars indicate mean ± SE. **(B)** Temperature dependency when (R)-CPP was administrated first and NBQX second (*n* = 5 animals). Other conventions are as in panel **(A)**.

Third, we investigated the effects of blocking glutamatergic excitatory inputs. We administered antagonists of the ionotropic glutamate receptors AMPA and NMDA (NBQX or [R]-CPP, respectively; Experiment 6). When we first administered NBQX (Figure 4A, left) or (R)-CPP (Figure 4B, left), peak amplitudes decreased compared with the control. Amplitudes further decreased with the additional administration of (R)-CPP or NBQX, respectively. In both cases, amplitude was significantly reduced by the combined administration of the glutamate receptor antagonists (β_c_ = −0.231, *P* = 0.043; β_c_ = −0.246, *P* = 0.0013; Supplementary Tables 8, 9). No significant change in peak latency was observed with the single or combined administration from the control condition (Figures 4A,B, right; Supplementary Tables 10, 11). Taken together, inhibiting glutamatergic excitatory inputs decreased the amplitude without inverting the temperature dependency. These results suggest that excitatory and inhibitory inputs play different roles in establishing the temperature dependency of neural activity; indeed, the negative correlation became a positive one by blocking the inhibitory, but not the excitatory, inputs.

**Figure 5:**
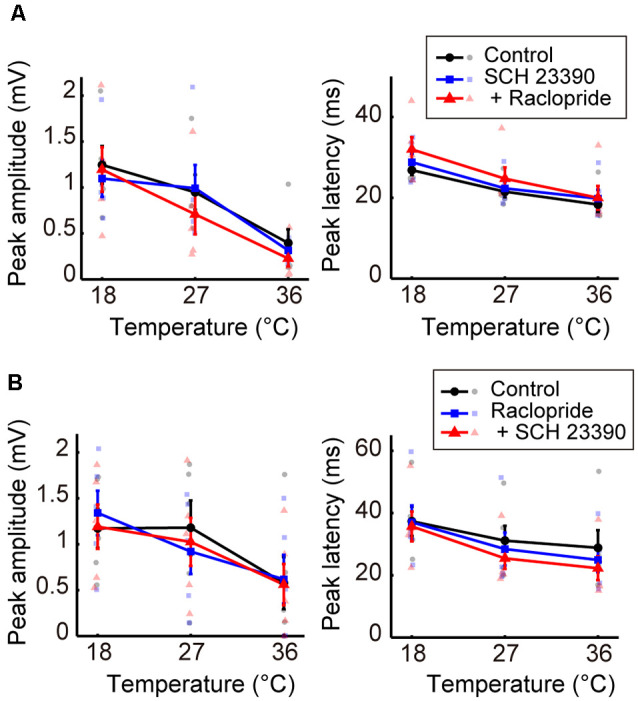
Effects of blocking dopaminergic inputs on the temperature dependency of evoked potentials (Experiment 7). **(A)** Temperature dependency when SCH 23390 was administered first and raclopride second (*n* = 5 animals). The peak amplitude (left) and the peak latency (right) of the evoked potentials are plotted against cortical temperature. Black, blue, and red symbols and lines indicate data for the control, for when one antagonist was administered, and for when two antagonists were administered, respectively. Error bars indicate mean ± SE. **(B)** Temperature dependency when raclopride was administrated first and SCH 23390 second (*n* = 5 animals). Other conventions are as in panel **(A)**.

We further examined the effects of dopaminergic inputs. In Experiment 7, dopamine receptor antagonists SCH 23390 hydrochloride (SCH 23390; D_1_/D_5_ receptor antagonist) and raclopride (D_2_/D_3_ receptor antagonist) were administered. Regardless of whether SCH 23390 or raclopride was administered first, no significant change in peak amplitude was observed in response to administering either the single or combined antagonists (Figures 5A,B, left; Supplementary Tables 12, 13). The peak latency consistently showed little change in response to these treatments (Figures 5A,B, right; Supplementary Tables 14, 15). The latency exhibited a small but significant decrease from the control condition only after the administration of raclopride followed by SCH 23390 (β_c_ = −7.109, *P* = 0.0031). Previous studies (Lavin et al., 2005; Kunori et al., 2014) showed that the dopamine antagonists did not change cortical activity evoked by stimulating the midbrain dopamine area at physiological temperature. Our data further revealed that modifying dopaminergic inputs does not cause a critical change in the temperature dependency of neural activity.

## Discussion

**Figure 6:**
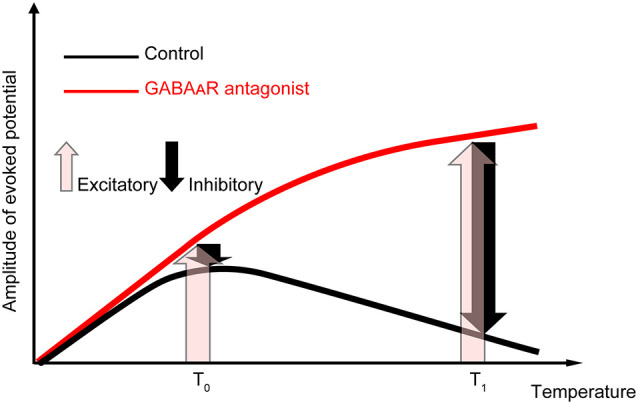
Explanatory diagram of the temperature dependency of evoked potentials. The evoked potentials in the control condition (black line) should result from the composition of excitatory inputs (pink upward arrows) and inhibitory inputs (black downward arrows). In contrast, when GABAergic inhibitory inputs are fully inactivated (red line), the net amplitude should result from the excitatory inputs. Our data suggest that the effect of temperature on inhibitory inputs is sufficiently large to overcome the excitatory inputs at higher temperatures.

Our data showed that amplitude and temperature have an inverted-U relationship in the control condition. When the local brain temperature decreased below 17°C, the amplitude of the evoked potential decreased to be nearly 0. In contrast, when the temperature decreased from near-physiological temperature (36°C), the amplitude of the evoked potential increased (>17°C). This raises a critical question: why does moderate cooling of the cortex away from physiological temperature increase the amplitude of evoked potentials? Our data indicate that the temperature dependency of evoked potentials is mainly determined by the temperature-dependent change in excitatory (i.e., glutamatergic) and inhibitory (i.e., GABAergic) inputs (Figure 6). When GABAergic inhibitory inputs are fully inactivated, the net amplitude should be derived from the excitatory inputs (pink arrows in Figure 6). Our data demonstrated that gabazine administration eliminated the negative correlation between amplitude and intermediate-to-high temperature, and the regression coefficients were positive. In contrast, when no antagonists are administered, the net amplitude should be determined mainly by both excitatory and inhibitory inputs (pink and black arrows in Figure 6, respectively). Since our data show that the net amplitudes were lower at higher temperatures, the effect of inhibitory inputs on the evoked potential amplitude may increase monotonically and be sufficiently large to override that of the excitatory inputs. Taken together, these results suggest that the balance of the effects of excitatory inputs and inhibitory inputs on evoked potentials are altered in a temperature-dependent manner, such that the smaller contribution of inhibitory inputs compared to excitatory inputs cumulatively generates an increased amplitude at a lower temperature. This could be due to the cumulative contribution of individual molecular processes (Korogod and Demianenko, 2017; see also Supplementary Discussion).

Previous behavioral studies have shown that focal brain cooling induces a reversible inactivation of various brain functions (Trendelenburg, 1911; Fuster and Bauer, 1974; Bauer and Fuster, 1976; Fuster et al., 1981; Payne et al., 1996; Hupé et al., 1998; Lomber and Malhotra, 2008). Those reports are consistent with our results of <17°C (i.e., left side of the inverted-U curve). At temperatures <17°C, the effects of inhibitory inputs would be minimal and those of excitatory inputs would decrease as the temperature decreases. From this perspective, the increase of evoked potential amplitudes due to cooling from the physiological temperature would be unexpected. However, some previous electrophysiological studies measuring field potentials are consistent with our data. Previous studies have shown, in anesthetized rats, that the amplitudes of cortical somatosensory evoked potentials increase when the cortical temperature decreases approximately 16°C from body temperature (Bindman et al., 1963; Schwerdtfeger et al., 1999). In addition, the amplitudes decreased when the cortical temperature increased 10°C from body temperature (Bindman et al., 1963). The increase of evoked potential amplitudes by decreasing the temperature has also been observed in the hippocampus. The peak amplitudes of field potentials in the dentate gyrus of rats when they were swimming at low temperatures (resulting in low hippocampal temperature) were larger than those when they were swimming at high temperatures (Moser et al., 1993). Aihara et al. (2001) measured the evoked potentials in the CA3 by stimulating the mossy fiber layer of the dentate gyrus in hippocampal slices in guinea pigs, and reported that the amplitudes of evoked potentials showed an inverted-U relationship with the temperature; the amplitude was the largest at approximately 31°C and was smaller at higher (31–37°C) and lower (15–31°C) ranges. Although the peak temperatures are quantitatively different among studies (possibly because different brain areas in different animals have their temperature characteristics), those data are qualitatively consistent with our present data in that the amplitudes increased when the cortical temperature decreased around the physiological temperature.

In this study, we focused on the largest peak of the evoked potentials because the primary purpose of the study was to elucidate the effects of temperature on neural information processing at the network level where multiple inputs are integrated. The latency of the largest peak at 36°C was ~25 ms in control conditions and ~50 ms in gabazine conditions (e.g., Figure 2C, right panel). Before the largest peak, there were a few small peaks of shorter latencies. In addition, the largest peak became smaller if smaller inter-stimulus intervals (~300 ms) were adopted (Supplementary Figure 1). Thus, the largest peaks that we analyzed were not regarded as mono- or di-synaptic activity triggered by midbrain stimulation. Instead, they result from polysynaptic local network activity involving multiple types of neurons. In addition to the initial components of the network activity (<~100 ms), subsequent oscillatory activities sometimes appeared after the peak activity that we analyzed (e.g., the right panel in Figure 2A). Those oscillations were observed mostly at higher temperatures in the gabazine conditions. How the temperature change affects the later oscillatory activities would be an interesting question to address in the future.

On the other hand, the smaller initial peaks may include mono- or di-synaptic transmissions. The dopamine neurons directly project to the frontal cortex (Emson and Koob, 1978; Bjorklund and Dunnett, 2007; Perez-Lopez et al., 2018), and they are known to co-release glutamate (Yamaguchi et al., 2011; Perez-Lopez et al., 2018). The glutamatergic (Yamaguchi et al., 2011) and GABAergic neurons (Carr and Sesack, 2000) in the VTA also project to the frontal cortex. The frontal cortex may also receive polysynaptic inputs from the VTA *via* different brain areas [e.g., *via* contralateral cortical areas (Kunori et al., 2014)]. Since previous studies that stimulated the VTA and recorded the evoked potentials in the frontal cortex under similar stimulation parameters to those in our study showed that the evoked potentials disappear when dopamine neurons are destroyed with 6-hydroxydopamine (Lavin et al., 2005; Watanabe et al., 2009; Kunori et al., 2014), orthodromic activation of dopamine neurons (either mono-synaptic or poly-synaptic) would play a key role in producing evoked activation in the frontal cortex. Although the inputs could not be precisely segregated from the evoked potentials recorded from the cortical surface, the temperature-modulation of each cortical input should be addressed in the future.

Temperature change affected not only the amplitudes of the evoked potentials but also their latencies. In contrast to amplitude, latency and temperature showed a negative, monotonic relationship, which was observed in both the control and the antagonist conditions. This could be because the related chemical processes are faster at higher temperatures, as shown by the Arrhenius equation (Cais et al., 2008). In the Arrhenius equation, the rate constant of reaction (*k*) is described as *k* = Ae^–E/RT^, where *E* is the activation energy, *R* is the gas constant, *T* is an absolute temperature, and *A* is a constant. Since evoked potentials are the result of a combination of chemical reactions, decreases in their rates can result in larger latencies of synaptic transmission (Sabatini and Regehr, 1996) and resultant delayed field potentials (Moser et al., 1993) at lower temperatures. Those changes in latencies could eventually cause a behavioral change. In fact, it has been reported that cooling the HVC (hyperstriatum ventrale pars caudalis, or high vocal center) slows the speed of songs in songbirds (Long and Fee, 2008).

When GABAergic inhibitory inputs were blocked, the peak latency increased. Experiment 5 showed that an increase in gabazine concentration causes an increase in latency. Previous studies using intracellular recording (Bernardi et al., 1982) and optical imaging with a voltage-sensitive dye (Kunori et al., 2014) have shown that midbrain stimulation first induces excitatory postsynaptic potentials (EPSPs), followed by inhibitory postsynaptic potentials (IPSPs) in frontal neurons. The chain of the cortical postsynaptic potential is characterized as an EPSP-IPSP sequence. In the present study, the later inactivation of IPSPs by a GABA_A_ receptor antagonist may have extended EPSPs and increased the peak latency.

What is the role of temperature dependency in the physiological brain? One possibility could be that the negative correlation between local brain temperature and amplitudes of evoked potentials contributes to neurovascular coupling. The increase in neural activity could induce increases in blood flow. MRI studies have shown that an increase in LFPs rather than in neuronal firings results in an increased BOLD signal (Logothetis et al., 2001). Since arterial blood temperature is lower than brain temperature (in animals without carotid rete; Abrams et al., 1965; Hayward and Baker, 1969; Kiyatkin et al., 2002; Kiyatkin, 2010), an increase in blood flow decreases local brain temperature (Hayward and Baker, 1969). Our data showed that a decrease in local brain temperature resulted in increased evoked potentials. This increase in LFPs could induce further increases in blood flow. Such amplification may contribute to the effective and flexible enhancement of neural activity and blood flow in specific brain regions.

In the present study, we recorded cortical field potentials in anesthetized animals. Although anesthesia can minimize the change in neural activity, metabolism, and consequent temperature fluctuation during experiments, our data could not determine how the local brain temperature modulates neuronal firing and animals’ behavior. Previous studies have shown that increases in local brain temperature as small as <2°C by light stimulation from an optical fiber decrease neuronal firing in some brain regions (Ait Ouares et al., 2019; Owen et al., 2019). Light delivery in the dorsal striatum induced a biased rotational behavior in a direction ipsilateral to the illumination, which could be explained by the decreased neuronal firing of the medium spiny neurons in the illuminated areas. Another study showed the opposite results, i.e., that heating the medial prefrontal cortex increased the neuronal firing in the illuminated area (Stujenske et al., 2015). An electrophysiological study in cats showed that cooling the cortex at 27–29°C induced a seizure followed by a silence of unit activities while cooling it at 19–21°C induced only a silence (Moseley et al., 1972). How the neuronal firing and the animals’ behavior are modulated by the local brain temperature and how they are related to the modulated field potentials should be addressed in the future.

In this study, we recorded cortical evoked potentials while controlling the local temperature. The amplitudes were negatively correlated with local cortical temperatures >17°C, but this negative correlation was eliminated by the administration of a GABA_A_ receptor antagonist. These results suggest that the negative correlation between the amplitudes of evoked potentials and the local temperature is caused by an alteration of the balance of contribution between excitatory and inhibitory inputs to the evoked potentials, possibly due to higher temperature sensitivity of inhibitory inputs. Although further investigation is necessary to elucidate how the temperature dependency of excitatory and inhibitory inputs influences brain functions, including cognitive and behavioral aspects, our present study indicates possibilities for exploring the mechanism underlying the mediation of neural information processing by temperature.

## Data Availability Statement

The datasets generated in the current study are available from the corresponding author upon reasonable request.

## Ethics Statement

The animal study was reviewed and approved by the Animal Care and Use Committee of the National Institute of Advanced Industrial Science and Technology (AIST).

## Author Contributions

MG, IT, and SY designed the research. MG, KN, MN, IT, and SY performed the experiments. MG and SY analyzed data. MG, MN and SY wrote the article. All of the authors discussed the results and commented on the manuscript. All authors contributed to the article and approved the submitted version.

## Conflict of Interest

The authors declare that the research was conducted in the absence of any commercial or financial relationships that could be construed as a potential conflict of interest.
