# Supplementary material for: Brain Temperature Alters Contributions of Excitatory and Inhibitory Inputs to Evoked Field Potentials in the Rat Frontal Cortex
Source: Front Cell Neurosci. 2020 Dec 7;14:593027. doi: 10.3389/fncel.2020.593027 (PMC7750431; doi:10.3389/fncel.2020.593027)
Supplement: Supplementary file 1 [file Data_Sheet_1.PDF]

Supplementary Material

Supplementary Table 1. Multiple regression analysis of peak amplitude in Experiments 1 and 3 (> 17°C).

		Coefficient	SE	*t*-stat	*P*-value
	Animal #2	0.710	0.162	4.395	2.0×10^{-5}
	Animal #3	–0.043	0.158	–0.272	0.79
	Animal #4	0.232	0.159	1.457	0.15
	Animal #5	0.564	0.162	3.494	6.2×10^{-4}
	Animal #6	0.936	0.159	5.869	2.5×10^{-8}
	Animal #7	1.362	0.144	9.429	5.0×10^{-17}
	Animal #8	0.873	0.140	6.239	3.9×10^{-9}
Control = (0)					
	T	–0.027	0.010	–2.721	0.0072
	Gabazine	2.341	0.148	15.824	2.1×10^{-34}
	T × Gabazine	0.092	0.014	6.699	3.5×10^{-10}
	Intercept	–0.254	0.150	–1.687	0.094
Gabazine = (0)					
	T	0.065	0.010	6.795	2.1×10^{-10}
	Control	–2.341	0.148	–15.824	2.1×10^{-34}
	T × Control	–0.092	0.014	–6.699	3.5×10^{-10}
	Intercept	2.088	0.151	13.844	4.8×10^{-29}

Model summary statistics: Number of observations = 169, Adjusted $R^2 = 0.795$, $F(10, 158) = 66.1$, $P = 2.77 \times 10^{-51}$ (versus constant model)

Supplementary Table 2. Multiple regression analysis of peak amplitude in Experiments 1 and 3 (< 17°C).

		Coefficient	SE	*t*-stat	*P*-value
	Animal #2	0.153	0.144	1.066	0.29
	Animal #3	0.069	0.152	0.452	0.65
	Animal #4	0.121	0.148	0.818	0.42
	Animal #5	0.356	0.144	2.467	0.017
	Animal #6	0.498	0.147	3.389	0.0013
	Animal #7	0.596	0.152	3.933	2.3×10^{-4}
	Animal #8	0.556	0.138	4.034	1.7×10^{-4}
Control = (0)					
	T	0.088	0.017	5.150	3.5×10^{-6}
	Gabazine	–0.479	0.540	–0.887	0.38
	T × Gabazine	–0.017	0.023	–0.741	0.46
	Intercept	2.317	0.399	5.809	3.1×10^{-7}
Gabazine = (0)					
	T	0.071	0.016	4.546	3.0×10^{-5}
	Control	0.479	0.540	0.887	0.38
	T × Control	0.017	0.023	0.741	0.46
	Intercept	1.838	0.382	4.813	1.2×10^{-5}

Model summary statistics: Number of observations = 67, Adjusted $R^2 = 0.564$, $F(10, 56) = 9.53$, $P = 5.05 \times 10^{-9}$ (versus constant model)

Supplementary Table 3. Multiple regression analysis of peak latency in Experiments 1 and 3.

		Coefficient	SE	*t*-stat	*P*-value
	Animal #2	–13.170	5.022	–2.623	0.0093
	Animal #3	–38.213	5.019	–7.613	7.3×10^{-13}
	Animal #4	–10.836	5.020	–2.159	0.032
	Animal #5	–35.622	5.018	–7.098	1.6×10^{-11}
	Animal #6	–9.312	5.018	–1.856	0.065
	Animal #7	–19.142	4.687	–4.084	6.2×10^{-5}
	Animal #8	–19.984	4.459	–4.482	1.2×10^{-5}
Control = (0)					
	T	–1.862	0.211	–8.827	3.1×10^{-16}
	Gabazine	26.534	4.482	5.920	1.2×10^{-8}
	T × Gabazine	–0.258	0.291	–0.888	0.38
	Intercept	37.791	4.699	8.043	5.0×10^{-14}
Gabazine = (0)					
	T	–2.120	0.201	–10.530	2.4×10^{-21}
	Control	–26.534	4.482	–5.920	1.2×10^{-8}
	T × Control	0.258	0.291	0.888	0.38
	Intercept	64.326	4.686	13.727	1.5×10^{-31}

Model summary statistics: Number of observations = 236, Adjusted R^2 = 0.664, $F(10, 225) = 47.5$, $P = 5.70 \times 10^{-50}$ (versus constant model)

Supplementary Table 4. Multiple regression analysis of peak amplitude in Experiments 2 and 4.

		Coefficient	SE	*t*-stat	*P*-value
	Animal #2	–0.884	0.253	–3.488	6.4×10^{-4}
	Animal #3	0.123	0.253	0.487	0.63
	Animal #4	–1.252	0.253	–4.944	2.0×10^{-6}
	Animal #5	–0.846	0.253	–3.338	0.0011
	Animal #6	0.299	0.253	1.179	0.24
	Animal #7	–1.122	0.253	–4.430	1.8×10^{-5}
	Animal #8	0.082	0.253	0.323	0.75
	Animal #9	0.237	0.253	0.937	0.35
	Animal #10	–0.759	0.253	–2.995	0.0032
	Animal #11	–1.374	0.253	–5.422	2.3×10^{-7}
	Animal #12	0.299	0.253	1.181	0.24
Control = (0)					
	T	–0.054	0.012	–4.427	1.8×10^{-5}
	Gabazine	2.425	0.186	13.007	1.6×10^{-26}
	T × Gabazine	0.083	0.017	4.823	3.4×10^{-6}
	Intercept	1.106	0.216	5.111	9.4×10^{-7}
Gabazine = (0)					
	T	0.029	0.012	2.395	0.018
	Control	–2.425	0.186	–13.007	1.6×10^{-26}
	T × Control	–0.083	0.017	–4.823	3.4×10^{-6}
	Intercept	3.531	0.216	16.321	2.5×10^{-35}

Model summary statistics: Number of observations = 168, Adjusted R^2 = 0.717, $F(14, 153) = 26.7$, $P = 8.26 \times 10^{-38}$ (versus constant model)

Supplementary Table 5. Multiple regression analysis of peak latency in Experiments 2 and 4.

		Coefficient	SE	*t*-stat	*P*-value
	Animal #2	27.350	2.877	9.506	4.0×10^{-17}
	Animal #3	7.013	2.877	2.437	0.016
	Animal #4	–2.744	2.877	–0.954	0.34
	Animal #5	1.258	2.877	0.437	0.66
	Animal #6	32.475	2.877	11.287	7.1×10^{-22}
	Animal #7	–0.181	2.877	–0.063	0.95
	Animal #8	2.580	2.877	0.897	0.37
	Animal #9	–1.200	2.877	–0.417	0.68
	Animal #10	–1.281	2.877	–0.445	0.66
	Animal #11	4.289	2.877	1.491	0.14
	Animal #12	2.832	2.877	0.984	0.33
Control = (0)					
	T	–0.932	0.138	–6.734	3.1×10^{-10}
	Gabazine	24.256	2.118	11.454	2.5×10^{-22}
	T × Gabazine	–0.797	0.196	–4.069	7.5×10^{-5}
	Intercept	17.173	2.457	6.990	8.0×10^{-11}
Gabazine = (0)					
	T	–1.729	0.138	–12.489	4.0×10^{-25}
	Control	–24.256	2.118	–11.454	2.5×10^{-22}
	T × Control	0.797	0.196	4.069	7.5×10^{-5}
	Intercept	41.429	2.457	16.862	1.0×10^{-36}

Model summary statistics: Number of observations = 168, Adjusted $R^2 = 0.883$, $F(14, 153) = 90.7$, $P = 1.51 \times 10^{-66}$ (versus constant model)

Supplementary Table 6. Multiple regression analysis of peak amplitude in Experiment 5.

		Coefficient	SE	*t*-stat	*P*-value
	Animal #2	–0.967	0.241	–4.017	1.7×10^{-4}
	Animal #3	–0.091	0.241	–0.378	0.71
	Animal #4	0.073	0.241	0.304	0.76
	Animal #5	–0.371	0.241	–1.540	0.13
	Animal #6	–0.204	0.241	–0.846	0.40
Control = (0, 0, 0)					
	T	–0.048	0.019	–2.517	0.015
	10 μM	0.896	0.311	2.883	0.0055
	100 μM	2.753	0.311	8.855	2.0×10^{-12}
	1,000 μM	3.520	0.311	11.322	2.0×10^{-16}
	T × 10 μM	–0.001	0.027	–0.036	0.97
	T × 100 μM	0.077	0.027	2.874	0.0056
	T ×1,000 μM	0.101	0.027	3.760	3.9×10^{-4}
	Intercept	0.418	0.269	1.551	0.13
10 μM = (0, 0, 0)					
	T	–0.049	0.019	–2.567	0.013
	Control	–0.896	0.311	–2.883	0.0055
	100 μM	1.857	0.311	5.972	1.4×10^{-7}
	1,000 μM	2.624	0.311	8.439	9.9×10^{-12}
	T × Control	0.001	0.027	0.036	0.97
	T × 100 μM	0.078	0.027	2.909	0.0051
	T ×1,000 μM	0.102	0.027	3.796	3.5×10^{-4}
	Intercept	1.314	0.269	4.880	8.4×10^{-6}
100 μM = (0, 0, 0)					
	T	0.029	0.019	1.547	0.13
	Control	–2.753	0.311	–8.855	2.0×10^{-12}
	10 μM	–1.857	0.311	–5.972	1.4×10^{-7}
	1,000 μM	0.767	0.311	2.467	0.017
	T × Control	–0.077	0.027	–2.874	0.0056
	T × 10 μM	–0.078	0.027	–2.909	0.0051
	T ×1,000 μM	0.024	0.027	0.887	0.38
	Intercept	3.171	0.269	11.777	4.0×10^{-17}
1,000 μM = (0, 0, 0)					
	T	0.053	0.019	2.801	0.0069
	Control	–3.520	0.311	–11.322	2.0×10^{-16}
	10 μM	–2.624	0.311	–8.439	9.9×10^{-12}
	100 μM	–0.767	0.311	–2.467	0.017
	T × Control	–0.101	0.027	–3.760	3.9×10^{-4}
	T × 10 μM	–0.102	0.027	–3.796	3.5×10^{-4}
	T ×100 μM	–0.024	0.027	–0.887	0.38
	Intercept	3.938	0.269	14.625	2.8×10^{-21}

Model summary statistics: Number of observations = 72, Adjusted $R^2 = 0.778$, $F(12, 59) = 21.8$, $P = 2.44 \times 10^{-17}$ (versus constant model)

Supplementary Table 7. Multiple regression analysis of peak latency in Experiment 5.

		Coefficient	SE	*t*-stat	*P*-value
	Animal #2	2.929	3.706	0.790	0.43
	Animal #3	13.899	3.706	3.750	4.0×10^{-4}
	Animal #4	7.601	3.706	2.051	0.045
	Animal #5	7.328	3.706	1.977	0.053
	Animal #6	22.361	3.706	6.033	1.1×10^{-7}
Control = (0, 0, 0)					
	T	–0.557	0.291	–1.912	0.061
	10 µM	10.637	4.785	2.223	0.030
	100 µM	21.934	4.785	4.584	2.4×10^{-5}
	1,000 µM	26.494	4.785	5.537	7.5×10^{-7}
	T × 10 µM	0.047	0.412	0.115	0.91
	T × 100 µM	–0.544	0.412	–1.321	0.19
	T × 1,000 µM	–1.098	0.412	–2.665	0.0099
	Intercept	14.350	4.144	3.463	0.0010
10 µM = (0, 0, 0)					
	T	–0.509	0.291	–1.749	0.085
	Control	–10.637	4.785	–2.223	0.030
	100 µM	11.297	4.785	2.361	0.022
	1,000 µM	15.857	4.785	3.314	0.0016
	T × Control	–0.047	0.412	–0.115	0.91
	T × 100 µM	–0.591	0.412	–1.436	0.16
	T × 1,000 µM	–1.145	0.412	–2.780	0.0073
	Intercept	24.987	4.144	6.030	1.2×10^{-7}
100 µM = (0, 0, 0)					
	T	–1.101	0.291	–3.780	3.7×10^{-4}
	Control	–21.934	4.785	–4.584	2.4×10^{-5}
	10 µM	–11.297	4.785	–2.361	0.022
	1,000 µM	4.560	4.785	0.953	0.34
	T × Control	0.544	0.412	1.321	0.19
	T × 10 µM	0.591	0.412	1.436	0.16
	T × 1,000 µM	–0.554	0.412	–1.345	0.18
	Intercept	36.284	4.144	8.757	2.9×10^{-12}
1,000 µM = (0, 0, 0)					
	T	–1.654	0.291	–5.681	4.3×10^{-7}
	Control	–26.494	4.785	–5.537	7.5×10^{-7}
	10 µM	–15.857	4.785	–3.314	0.0016
	100 µM	–4.560	4.785	–0.953	0.34
	T × Control	1.098	0.412	2.665	0.0099
	T × 10 µM	1.145	0.412	2.780	0.0073
	T ×100 µM	0.554	0.412	1.345	0.18
	Intercept	40.845	4.144	9.857	4.4×10^{-14}

Model summary statistics: Number of observations = 72, Adjusted R^2 = 0.788, $F(12, 59) = 22.9$, $P = 7.10 \times 10^{-18}$ (versus constant model)

Supplementary Table 8. Multiple regression analysis of peak amplitude in Experiment 6 (antagonist #1 = NBQX, antagonist #2 = (R)-CPP).

		Coefficient	SE	*t*-stat	*P*-value
	Animal #2	0.012	0.099	0.118	0.91
	Animal #3	0.006	0.099	0.061	0.95
	Animal #4	0.127	0.099	1.278	0.21
	Animal #5	–0.112	0.099	–1.133	0.26
	Animal #6	–0.067	0.099	–0.680	0.50
Control = (0, 0)					
	T	–0.059	0.007	–8.738	4.4×10^{-11}
	NBQX	–0.228	0.111	–2.063	0.045
	(R)-CPP	–0.231	0.111	–2.084	0.043
	T × NBQX	0.048	0.010	5.044	8.8×10^{-6}
	T × (R)-CPP	0.055	0.010	5.741	8.7×10^{-7}
	Intercept	0.234	0.101	2.311	0.026
NBQX = (0, 0)					
	T	–0.011	0.007	–1.605	0.12
	Control	0.228	0.111	2.063	0.045
	(R)-CPP	–0.002	0.111	–0.021	0.98
	T × Control	–0.048	0.010	–5.044	8.8×10^{-6}
	T × (R)-CPP	0.007	0.010	0.697	0.49
	Intercept	0.005	0.101	0.052	0.96
(R)-CPP = (0, 0)					
	T	–0.004	0.007	–0.619	0.54
	Control	0.231	0.111	2.084	0.043
	NBQX	0.002	0.111	0.021	0.98
	T × Control	–0.055	0.010	–5.741	8.7×10^{-7}
	T × NBQX	–0.007	0.010	–0.697	0.49
	Intercept	0.003	0.101	0.029	0.98

Model summary statistics: Number of observations = 54, Adjusted $R^2 = 0.796$, $F(10, 43) = 21.7$, $P = 1.08 \times 10^{-13}$ (versus constant model)

Supplementary Table 9. Multiple regression analysis of peak amplitude in Experiment 6 (antagonist #1 = (R)-CPP, antagonist #2 = NBQX).

		Coefficient	SE	t-stat	P-value
	Animal #2	0.241	0.057	4.185	1.8×10^{-4}
	Animal #3	0.090	0.057	1.574	0.12
	Animal #4	0.011	0.057	0.188	0.85
	Animal #5	0.214	0.057	3.730	6.8×10^{-4}
Control = (0, 0)					
	T	–0.040	0.004	–9.449	3.7×10^{-11}
	(R)-CPP	–0.141	0.070	–2.005	0.053
	NBQX	–0.246	0.070	–3.492	0.0013
	T × (R)-CPP	0.006	0.006	0.926	0.36
	T × NBQX	0.029	0.006	4.720	3.7×10^{-5}
	Intercept	0.130	0.062	2.113	0.042
(R)-CPP = (0, 0)					
	T	–0.035	0.004	–8.139	1.4×10^{-9}
	Control	0.141	0.070	2.005	0.053
	NBQX	–0.105	0.070	–1.487	0.15
	T × Control	–0.006	0.006	–0.926	0.36
	T × NBQX	0.023	0.006	3.793	5.7×10^{-4}
	Intercept	–0.011	0.062	–0.177	0.86
NBQX = (0, 0)					
	T	–0.012	0.004	–2.774	0.0088
	Control	0.246	0.070	3.492	0.0013
	(R)-CPP	0.105	0.070	1.487	0.15
	T × Control	–0.029	0.006	–4.720	3.7×10^{-5}
	T × (R)-CPP	–0.023	0.006	–3.793	5.7×10^{-4}
	Intercept	–0.116	0.062	–1.875	0.069

Model summary statistics: Number of observations = 45, Adjusted $R^2 = 0.877$, $F(9, 35) = 36.0$, $P = 4.28 \times 10^{-15}$ (versus constant model)

Supplementary Table 10. Multiple regression analysis of peak latency in Experiment 6 (antagonist #1 = NBQX, antagonist #2 = (R)-CPP).

		Coefficient	SE	*t*-stat	*P*-value
	Animal #2	1.800	2.626	0.686	0.50
	Animal #3	1.534	2.403	0.638	0.53
	Animal #4	–3.218	2.405	–1.338	0.19
	Animal #5	0.025	2.327	0.011	0.99
	Animal #6	–4.867	2.327	–2.092	0.044
Control = (0, 0)					
	T	–0.606	0.154	–3.930	3.7×10^{-4}
	NBQX	–0.070	2.661	–0.026	0.98
	(R)-CPP	–0.405	2.794	–0.145	0.89
	T × NBQX	0.052	0.219	0.239	0.81
	T × (R)-CPP	0.322	0.233	1.383	0.18
	Intercept	17.119	2.411	7.099	2.4×10^{-8}
NBQX = (0, 0)					
	T	–0.554	0.155	–3.581	0.0010
	Control	0.070	2.661	0.026	0.98
	(R)-CPP	–0.336	2.778	–0.121	0.90
	T × Control	–0.052	0.219	–0.239	0.81
	T × (R)-CPP	0.269	0.231	1.168	0.25
	Intercept	17.050	2.416	7.058	2.8×10^{-8}
(R)-CPP = (0, 0)					
	T	–0.284	0.174	–1.636	0.11
	Control	0.405	2.794	0.145	0.89
	NBQX	0.336	2.778	0.121	0.90
	T × Control	–0.322	0.233	–1.383	0.18
	T × NBQX	–0.269	0.231	–1.168	0.25
	Intercept	16.714	2.567	6.511	1.5×10^{-7}

Model summary statistics: Number of observations = 47, Adjusted $R^2 = 0.481$,
$F(10, 36) = 5.27$, $P = 9.46 \times 10^{-5}$ (versus constant model)

Supplementary Table 11. Multiple regression analysis of peak latency in Experiment 6 (antagonist #1 = (R)-CPP, antagonist #2 = NBQX).

		Coefficient	SE	t-stat	P-value
	Animal #2	–2.832	1.431	–1.979	0.056
	Animal #3	1.972	1.468	1.343	0.19
	Animal #4	–5.362	1.431	–3.747	7.1×10^{-4}
	Animal #5	0.323	1.462	0.221	0.83
Control = (0, 0)					
	T	–0.603	0.103	–5.869	1.6×10^{-6}
	(R)-CPP	0.326	1.688	0.193	0.85
	NBQX	–0.560	1.930	–0.290	0.77
	T × (R)-CPP	0.010	0.145	0.066	0.95
	T × NBQX	0.097	0.157	0.618	0.54
	Intercept	16.175	1.510	10.715	4.1×10^{-12}
(R)-CPP = (0, 0)					
	T	–0.593	0.103	–5.776	2.1×10^{-6}
	Control	–0.326	1.688	–0.193	0.85
	NBQX	–0.886	1.930	–0.459	0.65
	T × Control	–0.010	0.145	–0.066	0.95
	T × NBQX	0.088	0.157	0.557	0.58
	Intercept	16.502	1.510	10.931	2.5×10^{-12}
NBQX = (0, 0)					
	T	–0.506	0.119	–4.260	1.7×10^{-4}
	Control	0.560	1.930	0.290	0.77
	(R)-CPP	0.886	1.930	0.459	0.65
	T × Control	–0.097	0.157	–0.618	0.54
	T × (R)-CPP	–0.088	0.157	–0.557	0.58
	Intercept	15.615	1.855	8.419	1.3×10^{-9}

Model summary statistics: Number of observations = 42, Adjusted $R^2 = 0.740$, $F(9, 32) = 14.0$, $P = 8.78 \times 10^{-9}$ (versus constant model)

Supplementary Table 12. Multiple regression analysis of peak amplitude in Experiment 7 (antagonist #1 = SCH 23390, antagonist #2 = Raclopride).

		Coefficient	SE	*t*-stat	*P*-value
	Animal #2	–0.321	0.125	–2.558	0.015
	Animal #3	–0.233	0.125	–1.857	0.072
	Animal #4	0.693	0.125	5.535	3.2×10^{-6}
	Animal #5	–0.127	0.125	–1.015	0.32
Control = (0, 0)					
	T	–0.047	0.009	–5.060	1.3×10^{-5}
	SCH 23390	–0.027	0.153	–0.174	0.86
	Raclopride	–0.211	0.153	–1.376	0.18
	T × SCH 23390	0.004	0.013	0.292	0.77
	T × Raclopride	–0.007	0.013	–0.492	0.63
	Intercept	0.435	0.134	3.240	0.0026
SCH 23390 = (0, 0)					
	T	–0.043	0.009	–4.648	4.6×10^{-5}
	Control	0.027	0.153	0.174	0.86
	Raclopride	–0.185	0.153	–1.203	0.24
	T × Control	–0.004	0.013	–0.292	0.77
	T × Raclopride	–0.010	0.013	–0.784	0.44
	Intercept	0.409	0.134	3.041	0.0044
Raclopride = (0, 0)					
	T	–0.054	0.009	–5.757	1.6×10^{-6}
	Control	0.211	0.153	1.376	0.18
	SCH 23390	0.185	0.153	1.203	0.24
	T × Control	0.007	0.013	0.492	0.63
	T × SCH 23390	0.010	0.013	0.784	0.44
	Intercept	0.224	0.134	1.668	0.10

Model summary statistics: Number of observations = 45, Adjusted $R^2 = 0.781$, $F(9, 35) = 18.5$, $P = 8.00 \times 10^{-11}$ (versus constant model)

Supplementary Table 13. Multiple regression analysis of peak amplitude in Experiment 7 (antagonist #1 = Raclopride, antagonist #2 = SCH 23390).

		Coefficient	SE	*t*-stat	*P*-value
	Animal #2	1.071	0.124	8.652	3.2×10^{-10}
	Animal #3	1.427	0.124	11.522	1.8×10^{-13}
	Animal #4	0.235	0.124	1.894	0.066
	Animal #5	0.799	0.124	6.451	2.0×10^{-7}
Control = (0, 0)					
	T	–0.033	0.009	–3.580	0.0010
	Raclopride	–0.081	0.152	–0.532	0.60
	SCH 23390	–0.069	0.152	–0.457	0.65
	T × Raclopride	–0.007	0.013	–0.541	0.59
	T × SCH 23390	–0.002	0.013	–0.155	0.88
	Intercept	–0.027	0.133	–0.205	0.84
Raclopride = (0, 0)					
	T	–0.040	0.009	–4.345	1.1×10^{-4}
	Control	0.081	0.152	0.532	0.60
	SCH 23390	0.011	0.152	0.075	0.94
	T × Control	0.007	0.013	0.541	0.59
	T × SCH 23390	0.005	0.013	0.386	0.70
	Intercept	–0.108	0.133	–0.812	0.42
SCH 23390 = (0, 0)					
	T	–0.035	0.009	–3.799	5.6×10^{-4}
	Control	0.069	0.152	0.457	0.65
	Raclopride	–0.011	0.152	–0.075	0.94
	T × Control	0.002	0.013	0.155	0.88
	T × Raclopride	–0.005	0.013	–0.386	0.70
	Intercept	–0.097	0.133	–0.727	0.47

Model summary statistics: Number of observations = 45, Adjusted $R^2 = 0.832$, $F(9, 35) = 25.2$, $P = 9.35 \times 10^{-13}$ (versus constant model)

Supplementary Table 14. Multiple regression analysis of peak latency in Experiment 7 (antagonist #1 = SCH 23390, antagonist #2 = Raclopride).

		Coefficient	SE	*t*-stat	*P*-value
	Animal #2	0.960	1.299	0.739	0.46
	Animal #3	12.083	1.299	9.301	5.5×10^{-11}
	Animal #4	1.871	1.299	1.440	0.16
	Animal #5	3.259	1.299	2.508	0.017
Control = (0, 0)					
	T	–0.473	0.097	–4.883	2.3×10^{-5}
	SCH 23390	1.174	1.591	0.738	0.47
	Raclopride	1.603	1.591	1.007	0.32
	T × SCH 23390	–0.027	0.137	–0.199	0.84
	T × Raclopride	–0.192	0.137	–1.402	0.17
	Intercept	14.340	1.393	10.293	4.0×10^{-12}
SCH 23390 = (0, 0)					
	T	–0.500	0.097	–5.165	9.7×10^{-6}
	Control	–1.174	1.591	–0.738	0.47
	Raclopride	0.429	1.591	0.269	0.79
	T × Control	0.027	0.137	0.199	0.84
	T × Raclopride	–0.165	0.137	–1.203	0.24
	Intercept	15.514	1.393	11.136	4.7×10^{-13}
Raclopride = (0, 0)					
	T	–0.665	0.097	–6.867	5.7×10^{-8}
	Control	–1.603	1.591	–1.007	0.32
	SCH 23390	–0.429	1.591	–0.269	0.79
	T × Control	0.192	0.137	1.402	0.17
	T × SCH 23390	0.165	0.137	1.203	0.24
	Intercept	15.943	1.393	11.443	2.2×10^{-13}

Model summary statistics: Number of observations = 45, Adjusted $R^2 = 0.828$, $F(9, 35) = 24.6$, $P = 1.34 \times 10^{-12}$ (versus constant model)

Supplementary Table 15. Multiple regression analysis of peak latency in Experiment 7 (antagonist #1 = Raclopride, antagonist #2 = SCH 23390).

		Coefficient	SE	*t*-stat	*P*-value
	Animal #2	–9.175	1.828	–5.020	1.5×10^{-5}
	Animal #3	20.353	1.828	11.137	4.7×10^{-13}
	Animal #4	–2.317	1.828	–1.268	0.21
	Animal #5	–2.412	1.828	–1.320	0.20
Control = (0, 0)					
	T	–0.473	0.136	–3.471	0.0014
	Raclopride	–4.119	2.238	–1.840	0.074
	SCH 23390	–7.109	2.238	–3.176	0.0031
	T × Raclopride	–0.201	0.193	–1.042	0.30
	T × SCH 23390	–0.274	0.193	–1.420	0.16
	Intercept	26.900	1.960	13.726	1.2×10^{-15}
Raclopride = (0, 0)					
	T	–0.674	0.136	–4.945	1.9×10^{-5}
	Control	4.119	2.238	1.840	0.074
	SCH 23390	–2.990	2.238	–1.336	0.19
	T × Control	0.201	0.193	1.042	0.30
	T × SCH 23390	–0.073	0.193	–0.378	0.71
	Intercept	22.781	1.960	11.624	1.4×10^{-13}
SCH 23390 = (0, 0)					
	T	–0.746	0.136	–5.479	3.8×10^{-6}
	Control	7.109	2.238	3.176	0.0031
	Raclopride	2.990	2.238	1.336	0.19
	T × Control	0.274	0.193	1.420	0.16
	T × Raclopride	0.073	0.193	0.378	0.71
	Intercept	19.791	1.960	10.098	6.6×10^{-12}

Model summary statistics: Number of observations = 45, Adjusted $R^2 = 0.893$, $F(9, 35) = 41.9$, $P = 3.90 \times 10^{-16}$ (versus constant model)

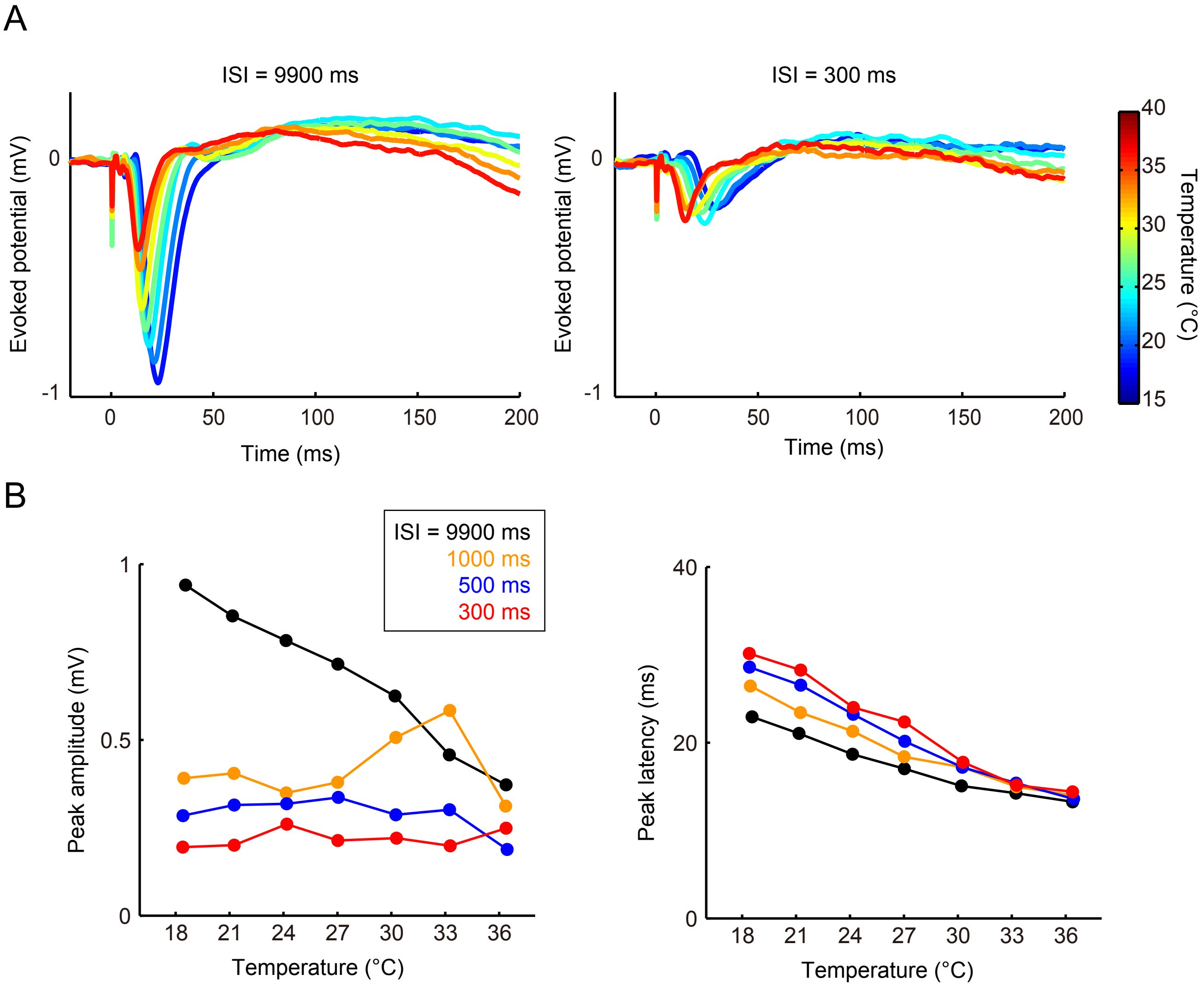

Supplementary Figure 1. Effects of inter-stimulus interval (ISI) on evoked potentials. To examine the effects of ISI on evoked potentials, we performed the same experiment as in Experiment 2 except that the ISI was changed (*n* = 1 animal). We adopted four ISIs of 9,900, 1,000, 500, and 300 ms for each cortical temperature. The cortical temperature increased from 18°C to 36°C in 3°C steps with body temperature regulation. At each temperature, we recorded the evoked potentials in 31 trials and data from 30 trials (from 2nd to 31st trials) were analyzed. **(A)** Waveforms of the evoked potentials. Waveforms for ISIs of 9,900 (left) and 300 ms (right) at each cortical temperature are represented by different colors, which are explained in the rightmost color bar. **(B)** Effects of ISI on the peak amplitude and peak latency of the evoked potentials. The peak amplitude (left) and the peak latency (right) for each ISI was plotted against the cortical temperature (*n* = 1 animal). Note that the evoked potentials could not follow short series of stimuli. The results support that the peak of the evoked potentials, which we focused on in the present study, may result at least partly from polysynaptic local network activity.

Supplementary Discussion

The modulation of the contributions of excitatory and inhibitory inputs to the evoked potentials could be due to the cumulative contribution of individual molecular processes. The conductance of ionotropic receptors such as the AMPA receptor (Postlethwaite et al., 2007), the NMDA receptor (Cais et al., 2008), and the $GABA_A$ receptor (De Koninck and Mody, 1994; Jenkins et al., 1999) increases at high temperatures. The majority of ion channels conducting depolarizing currents have been shown to increase their activity at high temperatures (Korogod and Demianenko, 2017). Some ion channels conducting hyperpolarizing currents such as the two-pore potassium channel (Korogod and Demianenko, 2017) and the inwardly rectifying potassium channel (Owen et al., 2019) are also known to increase their activities at high temperatures. Membrane transporters also exhibit thermal dependency. The activity of the Na^+-K^+ pump increases at high temperatures (Esmann and Skou, 1988), whereas that of the K^+-Cl^- cotransporter (KCC2) decreases (Hartmann and Nothwang, 2011). The mechanisms controlling intrasynaptic concentrations of neurotransmitters are also affected. For example, the rate of vesicular supply and release is accelerated at high temperatures (Pyott and Rosenmund, 2002), and transmitter uptake is also known to be dependent on temperature (Iversen and Neal, 1968; Asztely et al., 1997; Xie et al., 2000). Moreover, the diffusion coefficients of the neurotransmitters have been shown to increase as the temperature increases (Koike and Nagata, 1979). Taken together, the cumulative contribution of individual molecular processes may determine the temperature dependency of the net evoked potentials.

References

Asztely, F., Erdemli, G., and Kullmann, D.M. (1997). Extrasynaptic glutamate spillover in the hippocampus: dependence on temperature and the role of active glutamate uptake. *Neuron* 18(2)**,** 281-293. doi: 10.1016/s0896-6273(00)80268-8.

Cais, O., Sedlacek, M., Horak, M., Dittert, I., and Vyklicky, L., Jr. (2008). Temperature dependence of NR1/NR2B NMDA receptor channels. *Neuroscience* 151(2)**,** 428-438. doi: 10.1016/j.neuroscience.2007.11.002.

De Koninck, Y., and Mody, I. (1994). Noise analysis of miniature IPSCs in adult rat brain slices: properties and modulation of synaptic GABAA receptor channels. *J. Neurophysiol.* 71(4)**,** 1318-1335. doi: 10.1152/jn.1994.71.4.1318.

Esmann, M., and Skou, J.C. (1988). Temperature-dependencies of various catalytic activities of membrane-bound Na+/K+-ATPase from ox brain, ox kidney and shark rectal gland and of C12E8-solubilized shark Na+/K+-ATPase. *Biochem. Biophys. Acta* 944(3)**,** 344-350. doi: 10.1016/0005-2736(88)90504-4.

Hartmann, A.M., and Nothwang, H.G. (2011). Opposite temperature effect on transport activity of KCC2/KCC4 and N(K)CCs in HEK-293 cells. *BMC Res. Notes* 4**,** 526. doi: 10.1186/1756-0500-4-526.

Iversen, L.L., and Neal, M.J. (1968). The uptake of [3H]GABA by slices of rat cerebral cortex. *J. Neurochem.* 15(10)**,** 1141-1149. doi: 10.1111/j.1471-4159.1968.tb06831.x.

Jenkins, A., Franks, N.P., and Lieb, W.R. (1999). Effects of temperature and volatile anesthetics on GABA(A) receptors. *Anesthesiology* 90(2)**,** 484-491. doi: 10.1097/00000542-199902000-00024.

Koike, H., and Nagata, Y. (1979). Intra-axonal diffusion of [3H]acetylcholine and [3H]gamma-aminobutyric acid in a neurone of Aplysia. *J. Physiol.* 295**,** 397-417. doi: 10.1113/jphysiol.1979.sp012976.

Korogod, S.M., and Demianenko, L.E. (2017). Temperature effects on non-TRP ion channels and neuronal excitability. *Opera. Med. Physiol.* 3(3-4)**,** 84-92. doi: 10.20388/omp2017.003.0049.

Owen, S.F., Liu, M.H., and Kreitzer, A.C. (2019). Thermal constraints on in vivo optogenetic manipulations. *Nat. Neurosci.* 22(7)**,** 1061-1065. doi: 10.1038/s41593-019-0422-3.

Postlethwaite, M., Hennig, M.H., Steinert, J.R., Graham, B.P., and Forsythe, I.D. (2007). Acceleration of AMPA receptor kinetics underlies temperature-dependent changes in synaptic strength at the rat calyx of Held. *J. Physiol.* 579(Pt 1)**,** 69-84. doi: 10.1113/jphysiol.2006.123612.

Pyott, S.J., and Rosenmund, C. (2002). The effects of temperature on vesicular supply and release in autaptic cultures of rat and mouse hippocampal neurons. *J. Physiol.* 539(Pt 2)**,** 523-535. doi: 10.1113/jphysiol.2001.013277.

Xie, T., McCann, U.D., Kim, S., Yuan, J., and Ricaurte, G.A. (2000). Effect of temperature on dopamine transporter function and intracellular accumulation of methamphetamine: implications for methamphetamine-induced dopaminergic neurotoxicity. *J. Neurosci.* 20(20)**,** 7838-7845.
